# Analysis of AKAP7**γ** Dimerization

**DOI:** 10.1155/2015/371626

**Published:** 2015-08-31

**Authors:** Arpita Singh, Marc Rigatti, Andrew V. Le, Cathrine R. Carlson, Ion I. Moraru, Kimberly L. Dodge-Kafka

**Affiliations:** ^1^Pat and Jim Calhoun Center for Cardiology, Department of Cell Biology, University of Connecticut Health Center, Farmington, CT 06030, USA; ^2^The Richard D. Berlin Center for Cell Analysis & Modeling, University of Connecticut Health Center, Farmington, CT 06030, USA; ^3^Institute for Experimental Medical Research, Oslo University Hospital and University of Oslo, 0407 Oslo, Norway; ^4^KG Jebsen Cardiac Research Center and Center for Heart Failure Research, University of Oslo, Oslo, Norway

## Abstract

A-kinase anchoring proteins (AKAPs) constitute a family of scaffolding proteins that contribute to spatiotemporal regulation of PKA-mediated phosphorylation events. In particular, AKAP7 is a family of alternatively spliced proteins that participates in cardiac calcium dynamics. Here, we demonstrate via pull-down from transfected cells and by direct protein-protein association that AKAP7*γ* self-associates. Self-association appears to be an isoform specific phenomenon, as AKAP7*α* did not associate with itself or with AKAP7*γ*. However, AKAP7*γ* did associate with AKAP7*δ*, suggesting the long isoforms of the AKAP can form heterodimers. Surface plasmon resonance found that the AKAP7*γ* self-association occurs via two high affinity binding sites with *K*_*D*_ values in the low nanomolar range. Mapping of the binding sites by peptide array reveals that AKAP7*γ* interacts with itself through multiple regions. Photon counting histogram analysis (PCH) of AKAP7*γ*-EGFP expressed in HEK-293 cells confirmed that AKAP7*γ*-EGFP self-associates in a cellular context. Lastly, computational modeling of PKA dynamics within AKAP7*γ* complexes suggests that oligomerization may augment phosphorylation of scaffolded PKA substrates. In conclusion, our study reveals that AKAP7*γ* forms both homo- and heterodimers with the long isoforms of the AKAP and that this phenomenon could be an important step in mediating effective substrate phosphorylation in cellular microdomains.

## 1. Introduction

Phosphorylation of target proteins is a key mechanism utilized by the cell to induce changes in physiology and function. Numerous kinase-substrate interactions have been defined, yet the mechanism through which specificity of these phosphorylation events is achieved is still unclear. Recent work has suggested that the subcellular localization of signaling enzymes to discrete locations in the cell confers spatiotemporal control over phosphorylation events [1]. This is facilitated by scaffolding proteins, which tether the kinase to the same signaling complex as its substrates, allowing for increased speed and amplitude of phosphorylation events [2]. While the physiological significance of many kinase-binding scaffolds has been investigated, the influence of the molecular architecture of the complex on cellular processes is presently unclear.

Oligomerization of signaling enzymes is also an important mechanism for the transduction of cellular signals [3, 4]. Whether forming a hetero- or homooligomer, this process often begins at the level of the receptor as a first step in transducing a signal from outside the cell to the intracellular environment [5]. Oligomer formation is not limited to upstream signaling events. A few recent studies have demonstrated that scaffolding proteins can oligomerize, influencing their signaling functions. For example, the yeast scaffold Ste5, which initiates the mitogen-activated protein (MAP) kinase cascade in the* Saccharomyces cerevisiae* pheromone response pathway, dimerizes upon pheromone treatment, to enhance MAP kinase signaling in the cell [6]. Similarly, oligomerization of *β*-arrestin1 sequesters the scaffold in the cytoplasm, inhibiting its nuclear actions [7]. Importantly, these examples demonstrate that oligomerization greatly impacts the function of the scaffold.

For cAMP signaling, A-kinase anchoring proteins (AKAPs) focus the actions of the cAMP-dependent protein kinase (PKA) to specific targets, allowing for microdomains of kinase activity [2]. It is becoming evident that AKAPs have a more profound effect on the regulation of substrate phosphorylation than simply binding PKA. Many AKAPs have been shown to bind to phosphodiesterases (PDE), protein phosphatases (PP), and adenylyl cyclases (AC) as well, allowing for localized control of cAMP concentration [8–10]. Thus, AKAPs are able to coordinate all components necessary for PKA signaling, lending support to the concept of holistic substrate phosphorylation regulation by AKAPs. Importantly, several AKAPs have been found to oligomerize [11–14]. While the consequence of oligomerization has not been fully investigated, it is suggested that formation of these higher order structures increases the local concentration of kinases and phosphatase, allowing for increased, localized enzyme activity [12].

Our recent investigations focused on defining AKAP7-orchestrated cAMP microdomains. AKAP7 is a family of alternatively spliced isoforms that are known to play a role in cardiac calcium dynamics [15–20]. We have previously found that the long isoform AKAP7*γ* associates with Protein Kinase C (PKC), PP1, the PP1 inhibitor-1 (I-1), and phospholamban [16, 21–23] and others have shown that it interacts with both PDE4D3 and PDE3A [20, 24]. However, the molecular architecture of the signaling complex has not been investigated to date. Since oligomerization of AKAPs has been suggested to enhance enzyme activity, we were interested in determining if AKAP7 can self-associate and what effect this would have on its function. Here, we reveal that AKAP7*γ* oligomerizes, as demonstrated both* in vitro* and in a cellular context, and we predict via computational modeling that this may result in enhancement of PKA substrate phosphorylation. This self-interaction is limited to the large isoforms of AKAP7, correlating with previous findings that AKAP7*α* and AKAP7*γ* are differentially targeted in the cell and display unique functions [25]. Using peptide array to define the sites on AKAP7*γ* that mediate binding, we found several domains involved in the oligomerization. In agreement with this finding, surface plasmon resonance analysis (SPR) confirms that AKAP7*γ* interacts with itself via several high affinity binding sites. Using photon counting histogram (PCH) analysis we are able to detect dimerization of AKAP7*γ* in live cells. In order to investigate the functional significance of oligomerization on substrate phosphorylation, we developed a mathematical model using the Virtual Cell software (http://www.vcell.org). Computational analysis suggests that formation of dimeric complexes enhances the magnitude of substrate phosphorylation. Collectively, our work demonstrates that oligomerization of AKAP7*γ* impacts phosphorylation events orchestrated by the AKAP and suggests that these findings may be applied to other AKAP complexes.

## 2. Materials and Methods

### 2.1. Antibodies

For western blot analyses, the following antibodies were used: mouse monoclonal anti-GFP (Santa Cruz, 1 : 1000), goat polyclonal anti-dsRED (Santa Cruz, 1 : 1000), rabbit anti-His-tag (Millipore, 1 : 10,000), rabbit anti-mCherry (BioVision, 1 : 5000), and mouse anti-GST tag (Santa Cruz, 1 : 10,000). To immunoprecipitate EGFP-tagged proteins, rabbit anti-GFP (Santa Cruz, 3 *μ*g) or rabbit anti-mCherry (BioVision, 3 *μ*g) was used.

### 2.2. Construct Design

AKAP7*γ*-pET32a, AKAP7*α*-EGFP-N1, and AKAP7*γ*-EGFP-N1 were used as previously described [22, 25, 27]. AKAP7*γ* 1-150-EGFP, AKAP7*γ* 150-end-EGFP, and AKAP7*γ*-1-268-pET32a were constructed by PCR amplification and subcloning into the EcoRI-BamHI sites of pEGFP-N1 (Clontech) or the EcoRI-HindIII sites of pET32a (Millipore). AKAP7*γ*-pMAL was constructed by PCR amplification and subcloning into the BamHI-EcoRI sites of pMal-c5E (NEB). AKAP7*γ*-mCherry was constructed by PCR amplification and subcloning into the EcoRI-BamHI sites of pmCherry-C1 (Clontech). AKAP7*γ*-ΔPKA-EGFP was made by site directed mutagenesis by Genewiz. Monomeric EGFP and tandem EGFP used for PCH analysis were obtained from the lab of Dr. Kathy Herrick-Davis. The AKAP18*δ*-YFP construct was kindly provided by Prof. Enno Klussmann.

### 2.3. Recombinant Protein Purification

For purification of recombinant AKAP7*γ*-His/S-tag, bacteria were grown to OD_595_ 0.8 and then induced with IPTG (1 mM final concentration) for 3 hrs. After centrifugation, the bacteria were lysed in binding buffer (20 mM HEPES (pH 7.4), 0.5 M NaCl, and 5 mM imidazole along with 0.5% triton-X, 0.25 mg/mL lysozyme, 1 mM DTT, 1 mM EDTA, and protease inhibitor cocktail) for 2 hrs. Following centrifugation, protein was purified from supernatant by Ni-NTA agarose pulldown overnight (0.5 mL of 50% slurry, Ni-NTA solution, Qiagen). Ni-NTA agarose was washed 3x in binding buffer and protein was eluted with binding buffer + 800 mM imidazole. Protein was concentrated with Amicon ultra centrifugal filters (Millipore). Elution buffer was slowly exchanged with PBS while concentrating the protein in the centrifugal tubes. Protein concentration was determined by comparison to BSA standards via image densitometry of Coomassie stained SDS-PAGE gels. Image densitometry was performed with ImageJ software (NIH).

A similar protocol was followed for AKAP7*γ*-maltose binding protein (MBP) purification. The MBP-tagged protein was lysed in buffer containing protease inhibitor cocktail (20 mM Tris-HCl (pH 7.4), 200 mM NaCl, 1 mM EDTA, 1 mM Na azide, and 1 mM DTT). Separated cell lysate was passed through an amylose resin packed column, followed by 5 washes with lysis buffer. The protein was eluted with lysis buffer containing 10 mM maltose. The protein was concentrated and analyzed as described above.

### 2.4. Cell Transfection

For analysis of AKAP7*γ* binding to itself, HEK-293 cells at 50% confluency were transfected using calcium phosphate precipitation with pmCherry-AKAP7*γ* and either EGFP-AKAP7*γ* or EGFP control (10 *μ*g) overnight under 5% CO_2_ at 37°C. Cells were harvested 18 hrs later for cell extract. For analysis of AKAP7*γ* binding to AKAP7*δ*, HEK-293 cells at 50% confluency were transfected using calcium phosphate precipitation with AKAP7*γ*-YFP in the presence and absence of pmCherry-AKAP7*γ* (10 *μ*g) overnight under 5% CO_2_ at 37°C. Cells were harvested 18 hrs later for cell extract.

For PCH, HEK-293 cells were grown on uncoated glass coverslips to 70% confluency and transfected with 300 ng plasmid DNA (EGFP, tandem EGFP, or AKAP7*γ*-EGFP) in Opti-MEM (Invitrogen) serum-free medium using 6 *μ*L PLUS reagent and 4 *μ*L lipofectamine. After 2 hrs, transfection media were replaced with serum supplemented DMEM (10% FBS). Cells were used for photon counting histogram analysis 12–24 hrs after transfection.

Human aortic smooth muscle cells were obtained from Lonza and cultured according to the manufacturer's instructions.

### 2.5. Pulldown Assays

*In vitro*: purified His/S-tagged AKAP7*γ* (5 *μ*g) was incubated with either AKAP7*γ*-MBP (5 *μ*g) or MBP (5 *μ*g) and rocked overnight at 4°C. The next day, the amylose resin was added for 4 hrs to isolate the complex. The amylose beads were washed 4x in lysis buffer (as described in maltose tagged protein purification above) for 10–15 min while rocking. The components of the complex were then separated via SDS-PAGE and analyzed by western blot.

From cell supernatant: lysates prepared from transfected HEK-293 cells were incubated with 5 *μ*g purified recombinant protein preloaded on S-agarose beads (Novagen). For control groups, unrelated His/S-tag protein was used. The pulldowns were rocked overnight at 4°C, followed by 4 washes with their respective buffers for 10–15 min while rocking. The isolated complex was then subjected to separation via SDS-PAGE followed by western blot analysis.

### 2.6. SPR Analysis

SPR analysis was performed using the Biacore T100 platform. Purified recombinant AKAP7*γ* or AKAP7*α* was covalently immobilized to the surface of a sensor chip (Biacore type CM5) using NHS (*N*-hydroxysuccinimide) and EDC [1-ethyl-3-(3-dimethylaminopropyl)-carbodiimide] (Biacore amine coupling kit). The amount of ligand bound was 150 RU (resonance units). Protein analytes (AKAP7*γ* or AKAP7*α*) were diluted over a range of concentrations (12.5–200 nM) in HBS buffer (10 mM Hepes (pH 7.4), 150 mM NaCl, and 0.005% Surfactant P20) and were injected over the sensor surface at a flow rate of 30 *μ*L/min for 300 s. After injection phase, dissociation was monitored in HBS buffer for 300 s at the same flow rate. The surface was regenerated between injections using 10 mM NaCl at a flow rate of 50 *μ*L/min for 30 s. Obtained sensorgrams were analyzed by Biacore T100 evaluation software.

### 2.7. Peptide Array Membrane Synthesis and S-Tagged AKAP7*γ* Overlay

Human AKAP7*γ* was synthesized as 20-mer peptides with three amino acid offsets on membranes using a MultiPep automated peptide synthesizer (INTAVIS Bioanalytical Instruments AG, Koeln, Germany). The peptide array membranes were blocked for 2 hours in 1% casein in TBST (Tris-buffered saline with 1% tween) before incubation with 2 *µ*g/mL recombinant human S-tagged AKAP7*γ* in 1% casein (in TBST) overnight at 4°C. Thereafter the membranes were washed five times in TBST for 5 min before incubation with HRP-conjugated anti-S-tag antibody (Abcam, ab18589, 1 : 2500) for 1 hour at room temperature. Binding was detected using ECL Prime (RPN 2232, GE Healthcare) and chemiluminescence signals were detected by Las 1000 (Fujifilm, Tokyo, Japan).

For the peptide competition assay, the membranes were stripped for 45 min in 62.5 mM Tris-HCl, pH 6.8, 2% SDS, and 100 mM *β*-mercaptoethanol at 60°C and washed three times in TBST for 10 min before being blocked in 1% casein (in TBST) overnight at 4°C. Concomitantly, 2 *μ*g/mL of recombinant human His-S-AKAP18*γ* protein was preincubated with or without 10 *µ*M phospholamban peptide (1–30) overnight at 4°C, before being overlaid onto the peptide array membranes for 2 hours at room temperature. The membranes were washed, incubated with HRP-conjugated anti-S-tag antibody, and developed as described above. The PLB (amino acids 1–30) peptide was synthesized with an N-terminal biotin tag and purified to 89.0% purity by GenScript Corp., Piscataway, New Jersey, USA. Biotin-phospholamban (PLB) peptide amino acids 1–30: MEKVQYLTRSAIRRASTIEMPQQARQNLQN.

### 2.8. Photon Counting Histogram Analysis

Photon counting in live HEK-293 cells was performed with a Zeiss LSM 510 ConFocor 3 confocal microscope mounted on an Axiovert 200 M. Excitation was achieved at 488 nm with a 30 mW Argon laser at 50% power with attenuation set to 0.5. Fluorescence emission was collected with a 40x 1.2 NA water immersion objective (Zeiss C-Apochromat 1.2 W Corr UV-Vis-IR). Detection was performed with an avalanche photodiode (Zeiss). For each cell, five 10 s measurements were made from five different locations within a given cell. Photon counting histograms (PCH) were generated using LSM-FCS software with a bin time of 10 *μ*s. Global analysis of measurements with count rates ranging from 20 to 100 kHz was performed for each construct. Monomeric and dimeric (tandem) EGFP histograms were fit using a single-component model, while AKAP7*γ*-EGFP histograms were fit using a two-component model with the brightness values constrained to those derived from the fit of the monomeric and dimeric EGFP. Measurements exhibiting photo-bleaching or other anomalies producing instability of the fluctuation trace were excluded from analysis. A single-photon three-dimensional Gaussian model was assumed for the excitation volume.

### 2.9. Computational Modeling

A compartmental deterministic model of scaffold oligomerization was implemented using the Virtual Cell modeling software (http://www.vcell.org). The model is composed of 18 species, three compartments (plasma membrane, cytosol, and endoplasmic reticular membrane), and 11 reactions. The reaction network can be considered as being composed of four general modules: (1) production and degradation of cAMP, (2) the dynamic interactions of regulatory subunit, catalytic subunit and cAMP in the cytosol, (3) the dynamics of these same proteins and cAMP scaffolded on the ER membrane by AKAP, and (4) phosphorylation/dephosphorylation of target protein X on the ER membrane. A diagram of the reaction network and table of the kinetic parameters may be found in the supplemental information. For each oligomeric state of the scaffold represented in the different versions of the model, the concentration of each species is initialized with a value near steady state in unstimulated conditions. Each simulation is allowed to run for 200 s before stimulation, thus allowing each species to reach its equilibrium constant for each oligomer. Stimulation of PKA activity is achieved by increasing the amount of AC present on the plasma membrane from 5/*µ*m^2^ to 50/*µ*m^2^ for a period of 100 s, simulating the increase in Gs-associated AC during *β*-adrenergic stimulation. The resulting increase in cAMP leads to activation of scaffolded PKA and phosphorylation of the target protein X. Active PKA is then inactivated by interacting with the PKA inhibitor PKI. The ability of RII*α* to self-regulate via autophosphorylation has been shown previously [28]. We hypothesized that if a single catalytic subunit were to be activated within an AKAP complex, this may subsequently lead to activation of the remaining population of PKA within the complex. This hypothesis was implemented via (1). This equation adjusts for the probability of all catalytic subunits within the oligomeric complex becoming active following activation of a single catalytic subunit in the complex, where *n* represents the oligomeric state of the scaffold (e.g., 1 = monomer, 2 = dimer):

(1) $$\begin{array}{c} \left(\text{Total AKAP bound PKA}\right)\left(1-{\left(\frac{\text{Inactive}}{\text{Total}}\right)}^{n}\right)*n. \end{array}$$

The result of (1) represents the amount of catalytic subunit available to phosphorylate its target, *X*, and is substituted for [*E*] in the Henri-Michaelis Menten irreversible reaction step for phosphorylation of *X*:

(2) $$\begin{array}{c} V=\frac{\left[E\right]\cdot k_{cat}\cdot \left[X\right]}{K_{m}+\left[X\right]}. \end{array}$$

Simulations of all oligomeric states have the same amount of active AKAP-scaffolded PKA both at equilibrium and during stimulation. The behavior of the AKAP-scaffolded PKA is altered by (1) and (2) to reflect the hypothesized behavior in each oligomeric state. The full model is available via the Virtual Cell software in the public models folder for the username Marc.Rigatti.

## 3. Results

### 3.1. Direct Cellular Association of AKAP7*γ* with Itself

**Figure 1:**
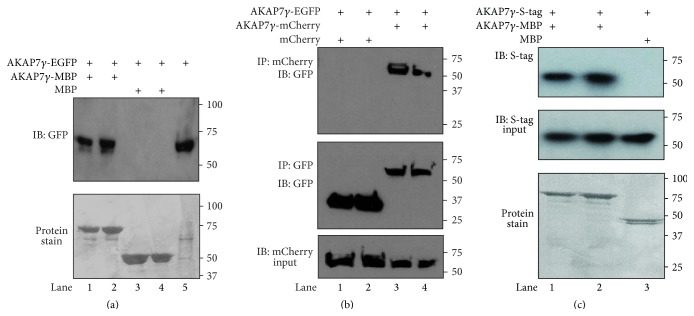
Direct cellular association of AKAP7*γ* with itself. (a) Lysates from AKAP7*γ*-EGFP transfected HEK-293 cells were subjected to pulldown assays using bacterially purified AKAP7*γ*-MBP (maltose binding protein) precharged on amylose agarose resin (lanes 1 and 2) or MBP precharged on amylose agarose resin (lanes 3 and 4). Input from the transfected cells (20 *μ*L) is shown in lane 5. Anti-GFP antibody was used for detecting protein interactions by western blot analysis (upper panel) while the protein stain of the nitrocellulose before western blot analysis is shown in the lower panel; *n* = 3. (b) mCherry western blot analysis of anti-GFP immunoprecipitates isolated from HEK-293 cells cotransfected with AKAP7*γ*-mCherry and either EGFP alone or AKAP7*γ*-EGFP (upper panel). To confirm immunoprecipitation of both EGFP and AKAP7*γ*-EGFP, the nitrocellulose membranes were stripped and reprobed for GFP using a monoclonal anti-GFP antibody (middle panel). Lower panel depicts mCherry western blot analysis of the input from each condition; *n* = 3. (c)* In vitro* pulldown of purified S-tagged AKAP7*γ* incubated with AKAP7*γ*-MBP precharged on amylose agarose resin (lanes 1 and 2) or control MBP (lane 3). Anti-His antibody was used to detect the interaction (upper panel). Equal amounts of S-tagged AKAP7*γ* were used in each condition, as shown by analysis of the input used in each experiment (middle panel). Lower panel shows the protein stain of the nitrocellulose before western blot analysis to demonstrate equal amounts of MBP-tagged proteins; *n* = 3.

In order to understand the molecular architecture of the AKAP7*γ* signaling complex and determine if the AKAP can be the basis for higher order structures, we first looked to see if purified, recombinant AKAP7*γ* could pull down an AKAP7*γ* complex when transiently expressed in cells. Lysate prepared from HEK-293 cells transfected with AKAP7*γ*-EGFP was incubated with amylose beads charged with either AKAP7*γ*-maltose binding protein (MBP) or MBP alone. The protein stain of the nitrocellulose before western blot analysis showing AKAP7*γ*-MBP (lanes 1 and 2) or MBP alone (lanes 3 and 4) is shown in Figure 1(a), lower panel. Input lysate (20 *μ*L) from cells expressing AKAP7*γ*-EGFP is shown in lane 5. Importantly, AKAP7*γ*-MBP was able to pull down AKAP7*γ*-EGFP from the transfected cell lysate (Figure 1(a), upper panel, lanes 1 and 2), suggesting that the AKAP7*γ* signaling complex may contain multiple AKAP7*γ* molecules. As a control, beads charged with purified MBP alone could not pull down AKAP7*γ*-EGFP (Figure 1(a), upper panel, lanes 3 and 4). To verify the existence of such complexes in cells, we coexpressed AKAP7*γ*-mCherry with either AKAP7*γ*-EGFP or EGFP alone in HEK-293 cells. AKAP7*γ*-mCherry input (20 *μ*L) for each immunoprecipitation is shown in Figure 1(b), lower panel. Immunoprecipitates from these cells isolated with an anti-GFP antibody and probed with mCherry demonstrate that AKAP7*γ*-mCherry and AKAP7*γ*-EGFP associate in cells (Figure 1(b), upper panel, lanes 3 and 4), while EGFP alone control does not coprecipitate AKAP7*γ*-mCherry (lanes 1 and 2). As a control, both EGFP (lanes 1 and 2) and AKAP7*γ*-EGFP (lanes 3 and 4) immunoprecipitates were probed with anti-GFP to demonstrate the immunoprecipitation of both proteins (Figure 1(b), middle panel). This coprecipitation could be due to multiple binding sites on other common binding partners within the AKAP complex. To determine if AKAP7*γ* can directly associate with itself, we conducted a pulldown assay using amylose beads charged with either bacterially expressed AKAP7*γ*-MBP (lanes 1 and 2) or MBP alone (lane 3) incubated with bacterially expressed S-tagged AKAP7*γ*. The protein stain of the nitrocellulose membrane before western blot analysis displaying the MPB tagged proteins used is shown in Figure 1(c), lower panel. S-tagged AKAP7*γ* input for each experiment is shown in the middle panel. Importantly, we detect a direct association between S-tagged AKAP7*γ* and AKAP7*γ*-MBP (lanes 1 and 2, upper panel), but not S-tagged AKAP7*γ* and MBP (lane 3, upper panel). Based on these experiments, we conclude that AKAP7*γ* can stably associate with itself.

### 3.2. Isoform Specificity and Binding Affinity of AKAP7*γ* Oligomers

**Figure 2:**
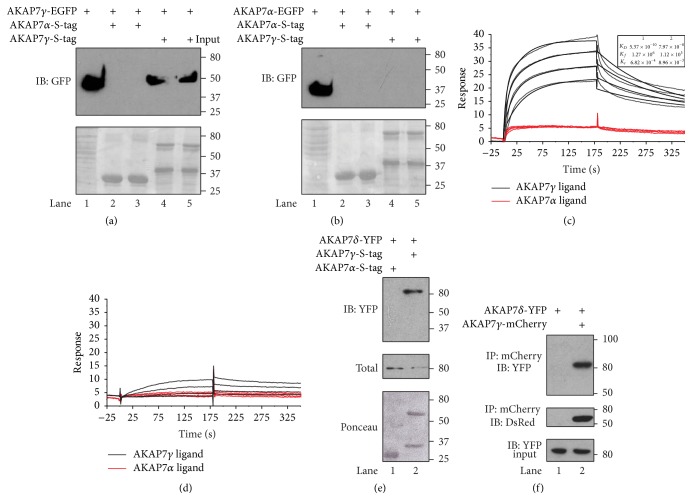
AKAP7*γ* forms homooligomers displaying high affinity interactions. (a) Lysates from AKAP7*γ*-EGFP transfected HEK-293 cells were subjected to pulldown assays using bacterially purified S-tagged AKAP7*α* (lanes 2 and 3) or AKAP7*γ* (lanes 4 and 5) precharged on S-protein resin. Anti-GFP antibody was used for detecting protein interactions by western blot analysis (upper panel). Protein stain of the nitrocellulose membrane before western blot analysis is shown in the lower panel. Input from the transfected cells (20 *μ*L) is shown in lane 1; *n* = 3. (b) Lysates from AKAP7*α*-EGFP transfected HEK-293 cells were subjected to pulldown assays using bacterially purified S-tagged AKAP7*α* (lanes 2 and 3) or AKAP7*γ* (lanes 4 and 5) precharged on S-protein resin. Anti-GFP antibody was used for detecting protein interactions by western blot analysis (upper panel). Protein stain of the nitrocellulose membrane before western blot analysis is shown in the lower panel. Input from the transfected cells (20 *μ*L) is shown in lane 1; *n* = 3. (c) SPR was performed by immobilizing AKAP7*γ* (150 RU, black) and AKAP7*α* (150 RU, red) in separate flow chambers on a CM5 chip and measuring the response when passing over a range of concentrations (25–200 nM) of either AKAP7*γ* (c) or AKAP7*α* (d). The sensorgram of AKAP7*γ* analyte binding to AKAP7*γ* ligand (c, black lines) was best fit with a heterogeneous analyte model suggesting the presence of two binding sites on AKAP7*γ*. The affinities of the two binding sites, as determined by the Biacore T100 evaluation software, were 0.537 nM and 79.7 nM with forward (*K*_*f*_) and reverse rates (*K*_*r*_) of association as shown. (e) Lysates from AKAP7*δ*-EGFP transfected HEK-293 cells were subjected to pulldown assays using bacterially purified S-tagged AKAP7*γ* (lane 1) or AKAP7*α* (lane 2) precharged on S-protein resin. Anti-YFP antibody was used for detecting protein interactions by western blot analysis (upper panel). Protein stain of the nitrocellulose membrane before western blot analysis is shown in the lower panel. Input from the transfected cells (20 *μ*L) is shown in middle panel; *n* = 3. (f) Western blot analysis of anti-mCherry immunoprecipitates isolated from HEK-293 cells cotransfected with AKAP7*δ*-YFP in the absence (lane 1) and presence (lane 2) of AKAP7*γ*-mCherry (upper panel). To confirm immunoprecipitation of AKAP7*γ*-mCherry, the nitrocellulose membranes were stripped and reprobed for mCherry using a goat polyclonal anti-DsRed antibody (middle panel). Lower panel depicts mCherry western blot analysis of the input from each condition; *n* = 3.

The* AKAP7* gene encodes at least 4 alternatively spliced isoforms: *α*, *β*, *γ*, and *δ* [25]. The smaller isoforms (*α*  and  *β*) are lipid modified in their N-terminal domain, directing their location to the plasma membrane [27]. However, the larger isoforms (*γ*  and  *δ*) require additional protein-protein interactions for their intracellular targeting [25]. We investigated whether the shorter isoforms could also associate with themselves or other isoforms. To address this question, lysate was prepared from HEK-293 cells transfected with AKAP7*γ*-EGFP and incubated with purified recombinant S-tagged AKAP7*α* or AKAP7*γ* bound to S-protein beads. The protein stain of the nitrocellulose membrane before western blot analysis, demonstrating that the beads are charged with AKAP7*α* (lanes 2 and 3) or AKAP*γ* (lanes 4 and 5), is shown in Figure 2(a), lower panel. Input lysate (20 *μ*L) from cells expressing AKAP7*γ*-EGFP is shown in lane 1. Importantly, S-tagged AKAP7*γ* was able to pull down AKAP7*γ*-EGFP specifically from the transfected cell lysate (Figure 2(a), upper panel, lanes 4 and 5). However, we did not detect the presence of AKAP7*γ*-EGFP with AKAP7*α* pulldowns (Figure 2(a), upper panel, lanes 2 and 3), suggesting that there is no cross isoform interaction. To further confirm this observation, we conducted the inverse experiment. Lysate collected from HEK-293 cells transfected with AKAP7*α*-EGFP was incubated with S-beads charged with S-tagged AKAP7*α* (lanes 2 and 3) or AKAP7*γ* (lanes 4 and 5). As expected, we did not detect any interaction between the two isoforms (Figure 2(b), upper panel).

To further increase our understanding of this interaction, the detailed binding kinetics of the AKAP7*γ* interaction was explored by surface plasmon resonance (SPR). AKAP7*γ* or AKAP7*α* was chemically conjugated in separate flow chambers on a CM5 sensor chip. A range of concentrations of either AKAP7*γ* (Figure 2(c)) or AKAP7*α* (Figure 2(d)) analyte (12.5 nM to 200 nM) was injected, and the binding dissociation constant (*K*_*D*_) was determined from the fit of the sensorgrams. We detected a high affinity interaction between AKAP7*γ* and itself that is best fit with a heterogeneous analyte model, suggesting at least two sites of interaction that display binding affinities of 0.537 nM and 79.7 nM (*χ* ^2^ = 1.33) (Figure 2(c), black lines). However, no significant binding between AKAP7*α* and AKAP7*γ* was detected (Figure 2(c), red lines). This was confirmed in reciprocal experiments, where we injected AKAP7*α* in the flow chambers conjugated with either AKAP7*γ* or AKAP7*α*. No binding of AKAP7*α* and AKAP7*γ* analyte to AKAP7*α* ligand was detected (Figure 2(d)).

Our data so far suggests that the long isoform of the AKAP can form homodimers. Next we investigated if AKAP7*γ* can associate with another long isoform of the AKAP7 family, AKAP7*δ*. These two splice variants differ in only their extreme N-terminus, suggesting that they may be able to associate through their common domains. To test this hypothesis, lysate from HEK293 cells transfected with AKAP7*δ*-YFP was incubated with purified recombinant S-tagged AKAP7*α* or AKAP7*γ* bound to S-protein beads. The protein stain of the nitrocellulose before western blot analysis demonstrating that the beads are charged with AKAP7*α* (lane 1) or AKAP*γ* (lane 2) is shown in Figure 2(e), lower panel. Input lysate (20 *μ*L) from cells expressing AKAP7*δ*-YFP is shown in the middle panel. Importantly, AKAP7*γ* was able to associate with AKAP7*δ*. To determine if these two isoforms could associate in cells, we coexpressed AKAP7*δ*-YFP in the presence (lane 2) and absence (lane 1) of AKAP7*γ*-mCherry HEK-293 cells. AKAP7*δ*-YFP input (20 *μ*L) for each immunoprecipitation is shown in Figure 2(f), lower panel. Immunoprecipitates from these cells isolated with an anti-mCherry antibody demonstrate that AKAP7*γ*-mCherry and AKAP7*δ*-YFP associate in cells (Figure 2(f), upper panel). As a control, the immunoprecipitates were also probed with anti-DsRed to demonstrate the immunoprecipitation of the AKAP7*γ*-mCherry (Figure 2(f), middle panel). However, AKAP7*γ* does not associate either mAKAP or AKAP79, other AKAPs that are expressed in the heart (Supplementary Figure 2 in Supplementary Material available online at http://dx.doi.org/10.1155/2015/371626) [29, 30]. Taken together, these data suggest that AKAP7*γ* can form both homodimers and heterodimers with the longer isoforms of the AKAP7 gene family.

### 3.3. Mapping the Sites in AKAP7*γ* Responsible for Association

**Figure 3:**
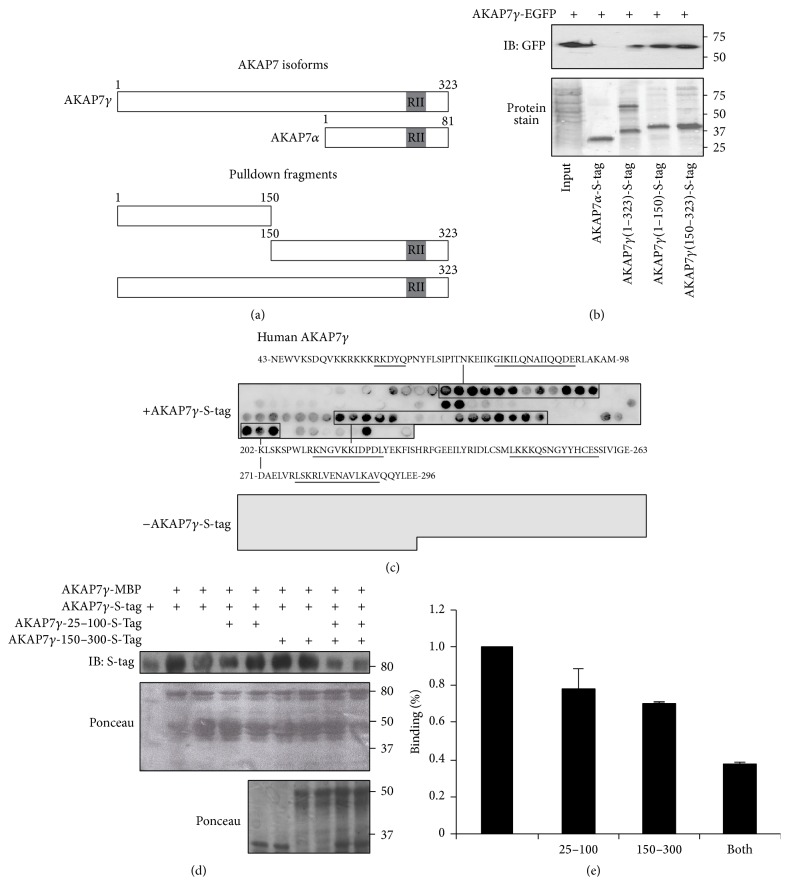
Mapping the sites in AKAP7*γ* responsible for oligomerization. (a) Schematic depicting AKAP7*γ* fragments used for pulldown experiments. (b) Lysates from AKAP7*γ*-EGFP transfected HEK-293 cells were subjected to pulldown assays using bacterially purified S-tagged AKAP7 deletion fragments precharged on S-protein resin; AKAP7*γ*-150–323, AKAP7*γ*-1–150, full length AKAP7*γ*, or AKAP7*α*. Anti-GFP antibody was used for detecting protein interactions by western blot analysis (upper panel). Protein stain of the nitrocellulose membrane before western blot analysis is shown in the lower panel. Input from the transfected cells (20 *μ*L) is shown in the first lane; *n* = 3. (c) The full length AKAP7*γ* sequence was spotted as overlapping 20-mer peptides with 3 amino acid shifts on cellulose membranes and subjected to overlay using S-tagged AKAP7*γ*. Binding was detected by chemiluminescence using an HRP-conjugated S-protein antibody (upper panel). Underlined sequences indicate regions of highest affinity binding. The antibody did not detect any binding in the absence of S-tagged AKAP7*γ* overlay (lower panel); *n* = 4. (d)* In vitro* pulldown of purified S-tagged AKAP7*γ* (10 *μ*g) incubated with AKAP7*γ*-MBP precharged on amylose agarose resin (lanes 2 through 9) with increasing concentrations of either AKAP7*γ*-1–100 (lanes 4-5), AKAP7*γ*-150–300 (lanes 6-7), or both (lanes 8-9). Anti-S-tag antibody was used to detect the interaction of AKAP7*γ* with AKAP7*γ*-MBP (upper panel). The competing fragments are shown in the lower panel. Middle panel shows the protein stain of the nitrocellulose before western blot analysis to demonstrate equal amounts of MBP-tagged proteins. (e) Normalization of binding shown in (d).

The SPR results suggest that multiple sites of interaction may exist between AKAP7*γ* and itself. To determine the number and location of binding sites, a series of AKAP7*γ* fragments were created. The common PKA binding domain is maintained, contained in amino acids 29–42 of AKAP18*α* (Figure 3(a)). These fragments were incubated with lysate from HEK-293 cells transfected with AKAP7*γ*-EGFP (Figure 3(b)). The protein stain of S-protein beads charged with the different AKAP7 proteins used in the experiment is shown in Figure 3(a), lower panel. Input lysate (20 *μ*L) from cells expressing AKAP7*γ*-EGFP is shown in the designated lane. In confirmation of our previous results, recombinant purified full length AKAP7*γ*, but not AKAP7*α*, was able to pull down AKAP7*γ*-EGFP from transfected cell lysate (Figure 3(b), upper panel). Additionally, both halves of AKAP7*γ*, AKAP7*γ*(1–150) and AKAP7*γ*(150–323), were able to pull down AKAP7*γ*-EGFP from transfected cell lysate, revealing that each half of the protein participates in the homoassociation of AKAP7*γ* (Figure 3(a)).

To further refine the location of the binding sites, human AKAP7*γ* was synthesized as 20-mer peptides with three amino acid offsets on membranes using a MultiPep automated peptide synthesizer and then subjected to overlay with bacterially purified, full length, S-tagged AKAP7*γ* (Figure 3(c), upper panel). Using an HRP-conjugated anti-S-tag antibody, binding was observed in multiple regions residing primarily in amino acids 43–98 and amino acids 202–296. Underlined sequences depict regions of strongest binding. Importantly, the HRP-conjugated anti-S-tag antibody did not detect any spots in the absence of overlay with purified AKAP7*γ* (Figure 3(c), lower panel). This data offers further confirmation that there are two regions of interaction between AKAP7*γ* and itself.

We next looked to see if any of these regions could be used to disrupt the oligomerization. We conducted a pulldown assay using amylose beads charged with bacterially expressed AKAP7*γ*-MBP incubated with bacterially expressed S-tagged AKAP7*γ* in the presence and absence of a competing peptide consisting of the regions shown by peptide array to be involved in orchestrating the oligomerization. The protein stain of the nitrocellulose membrane before western blot analysis displaying the MPB tagged proteins is shown in Figure 3(d), middle panel. S-tagged AKAP7*γ* input is shown in the first lane of the upper panel. Protein stain of the peptides used is shown in the lower panel. Competition using an untagged peptide consisting of amino acids 25–100 of AKAP7*γ* did not significantly disrupt binding (lanes 4-5). Furthermore, a peptide consisting of amino acids 150–300 of AKAP7*γ* also did not significantly reduce the presence of full length AKAP bound to the AKAP7*γ*-MBP (lanes 6-7). However, using both peptides simultaneously did attenuate oligomerization (lanes 8-9). Quantification of the percent binding is shown in Figure 3(e). Using just one competing peptide only reduced binding by 25% and 30%, respectively. However, the combination of both peptides reduced binding by over 65%. Taken together, the data shown in Figure 3 demonstrates that multiple sites of contact orchestrate the oligomerization of the AKAP.

### 3.4. PKA and PLB Binding to AKAP7*γ* Do Not Interfere with AKAP7*γ* Dimerization

**Figure 4:**
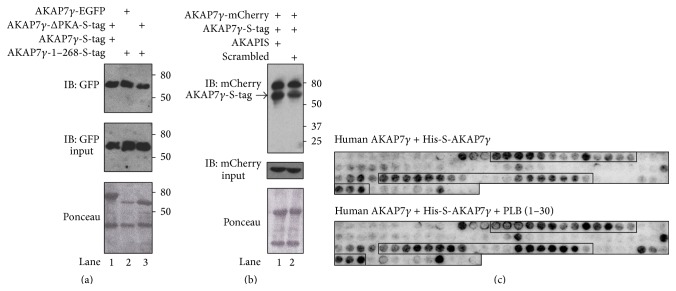
PKA and PLB binding to AKAP7*γ* do not interfere with AKAP7*γ* dimerization. (a) Lysates from either AKAP7*γ*-EGFP or AKAP7*γ*-ΔPKA-EGFP transfected HEK-293 cells were subjected to pulldown assays using bacterially purified S-tagged AKAP7*γ* proteins precharged on S-protein resin; full length AKAP7*γ* or AKAP7*γ*-1–268 (which lacks the PKA binding domain). Anti-GFP antibody was used for detecting protein interactions by western blot analysis (upper panel). Protein stain of the nitrocellulose membrane before western blot analysis is shown in the lower panel. Input from the transfected cells (20 *μ*L) is shown in the middle panel; *n* = 3. (b) Lysates from AKAP7*γ*-mCherry transfected HEK-293 cells were subjected to pulldown assays using bacterially purified S-tagged AKAP7*γ* precharged on S-protein resin. After an overnight incubation, the pulldowns were washed extensively and then incubated with the PKA anchoring disrupting peptide AKAPIS or control, scrambled peptide (10 *μ*M) for 3 hours. The pulldowns were washed again, and association of AKAP7*γ* was determined by anti-mCherry antibody (upper panel). Protein stain of the nitrocellulose membrane before western blot analysis is shown in the lower panel. Input from the transfected cells (20 *μ*L) is shown in the middle panel; *n* = 3. (c) The AKAP18*γ* peptide array membranes used in (c) were stripped for 45 min in 62.5 mM Tris-HCl, pH 6.8, 2% SDS, and 100 mM *β*-mercaptoethanol at 60°C and washed three times in TBST for 10 min before being blocked in 1% casein (in TBST) overnight at 4°C. Concomitantly, 2 *µ*g/mL of recombinant human His-S-AKAP18*γ* protein was preincubated with or without 10 *µ*M phospholamban peptide (1–30) overnight at 4°C, before being overlaid onto the peptide array membranes for 2 hours at room temperature. The membranes were washed, incubated with HRP-conjugated anti-S-tag antibody, and developed as described above.

Previous work has defined many AKAP7*γ* binding partners, including PKC, PP1, the PP1 inhibitor I-1, phospholamban, PDE4D3, and PDE3A [16, 20–24]. As both PKA and phospholamban form higher order structures, we investigated the role these binding domain may play in the oligomerization of AKAP7*γ*. To begin, HEK293 cells were transfected with either full length AKAP7*γ*-EGFP or the one lacking the PKA binding domain (AKAP7*γ*-ΔPKA-EGFP). Isolated lysate was incubated with purified recombinant S-tagged full length AKAP7*γ* or AKAP7*γ* lacking the PKA domain (AKAP7*γ*-1-268) bound to S-protein beads. The protein stain of the nitrocellulose before western blot analysis demonstrating that the beads are charged is shown in Figure 4(a), lower panel. Input lysate (20 *μ*L) from cells is shown in the middle panel. As shown in Figure 4(a), full length AKAP7*γ* was able to pull down AKAP7*γ*-ΔPKA-EGFP from cells (Figure 4(a), lane 1), suggesting that the PKA binding domain does not participate in the interaction. In validation of this, AKAP7*γ*-1-268-S-tag, which lacks the PKA binding domain, pull downs both AKAP7*γ*-EGFP and AKAP7*γ*-ΔPKA-EGFP. To further confirm this finding, we utilized a peptide that is known to disrupt PKA/AKAP interactions (AKAPIS) [31]. Lysate from HEK293 cells transfected with AKAP7*γ*-mCherry was incubated with purified recombinant S-tagged AKAP7*γ* bound to S-protein beads. The protein stain of the nitrocellulose before western blot analysis demonstrating that the beads are charged AKAP*γ* is shown in Figure 4(b), lower panel. Input lysate (20 *μ*L) from cells expressing AKAP7*γ*-mCherry is shown in the middle panel. Importantly, incubation of either AKAPIS or a control, scrambled peptide, did not disrupt AKAP7*γ* homoassociation.

Next we looked to see if the phospholamban binding domain, contained in amino acids 124–220, was involved in directing the interaction. Peptide array studies were conducted by overlaying either His-S-AKAP7*γ* or His-S-AKAP7*γ* and PLB (1–30) onto 20-mer AKAP7*γ* fragments as in Figure 3(c). The addition of a phospholamban peptide (1–30) containing the AKAP binding site did not significantly alter the binding pattern in the peptide array, indicating that PLB binding to AKAP7*γ* does not interfere with homodimerization. Taken together, the data presented in Figure 4 suggest that neither the PKA binding domain nor the PLB binding domains participate in the oligomerization of AKAP7*γ*.

### 3.5. Dimerization of AKAP7*γ* Can Be Detected in Cells

**Figure 5:**
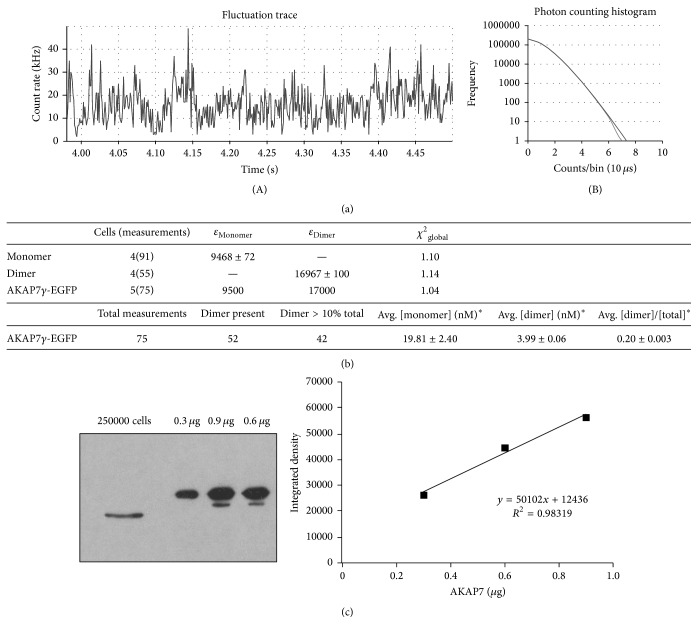
Photon counting histogram detects dimerization of AKAP7*γ* in live cells. (a) A representative 0.5 s trace is shown to demonstrate the fluctuation in intensity over time as particles enter and leave the measurement volume (A). The full 10 s fluctuation trace is divided into 10 *μ*s bins and plotted as a histogram of frequency versus counts/bin (B). Fitting of the histogram yields the concentration (number, *n*) and brightness (*ε*) of the fluorescent particles. (b) Data summary. HEK-293 cells were transfected with monomeric EGFP, dimeric EGFP, or AKAP7*γ*-EGFP. Selected cells were measured 5 times at 5 different positions within the cytosol for 10 s per measurement. Measurements exhibiting bleaching or other instabilities in fluorescence intensity were excluded from analysis. Global fitting of the data produced near ideal fits (*χ* ^2^_global_ of 1.0 is ideal). Monomeric and dimeric EGFP were fit using a single-component model, while AKAP7*γ* was fit using a 2-component model allowing for the detection of both monomeric and dimeric AKAP7*γ*-EGFP. ^*∗*^Average concentrations and [dimer]/[total] were determined from the 52 measurements in which the presence of dimer was detected. (c) Known quantities of purified AKAP7*γ*-S-tag were subjected to SDS-PAGE alongside lysate obtained from 250,000 human aortic smooth muscle cells. Using the standard curve created from densitometry measurements of the known quantities of AKAP7*γ*-S-Tag, we were able to estimate the amount of AKAP7*γ* per cell. The mass of AKAP7*γ* per cell was divided by its molecular weight (37 kD) to convert mass to moles and then divided by a cellular volume of 30 pL to arrive at a final cellular concentration of 180 nM. This volume is the approximate volume of cardiac myocytes, which we expect to be similar to that of an aortic smooth muscle cell [26].

The existence of two sites of interaction of AKAP7*γ* with itself suggests the potential to form multimeric structures. This possibility was investigated via photon counting histogram (PCH) analysis of AKAP7*γ*-EGFP expressed in live HEK-293 cells. Photon counting histogram analysis is a method of fluorescence correlation spectroscopy that allows for determination of the size (brightness, *ε*) and concentration (number, *n*) of given oligomer from fluctuations in signal intensity. This technique uses standard confocal optics to create a measurement volume on the order of 0.5 fL. As fluorescent particles diffuse through the measurement volume, their signal is detected by a highly sensitive detector, such as an avalanche photodiode. The change in signal intensity is dependent upon the average number of particles within the volume (*n*) and the brightness of a given particle (*ε*). At low average particle concentrations, the change in fluorescence intensity resulting from a single particle entering or leaving the volume is readily detected, while at high concentrations this change is less detectable. Detection of the change in intensity over time yields a fluctuation trace. The fluctuation trace is divided into small increments of time (bins) and the signal intensity (counts) is averaged within each increment. The average signal for each bin is then plotted as frequency versus counts/bin to yield the photon counting histogram (Figure 5(a)), which can be fit to determine the brightness and concentration of the fluorescent particles present. Cells expressing low yet detectable amounts of AKAP7*γ*-EGFP were selected for analysis due to the dependence of signal variance on the inverse of the number of particles present in the confocal volume. It is noteworthy that cells selected for analysis expressed AKAP7*γ*-EGFP in the nanomolar range, since higher levels of protein typically seen with overexpression cannot be reliably measured by FCS. For each cell chosen, five regions were selected from within the cytoplasm for analysis. Each region was measured 5 times for 10 s per measurement. The data for each construct was fit globally with either a single-component model (EGFP monomer and dimer) or a two-component model (AKAP7*γ*-EGFP) yielding a brightness value (*ε*) and concentration for either one or two species, respectively. This type of fitting allows for the derivation of a single brightness value based upon the aggregate data from all measurements of a given fluorescent construct, rather than averaging individual values derived from each individual measurement. Global fitting of the EGFP monomer and dimer yielded brightness values of 9468 ± 72 (*χ* ^2^_reduced_ = 1.10) and 16967 ± 100 (*χ* ^2^_reduced_ = 1.14), respectively (Figure 5(b)). For AKAP7*γ*-EGFP 75 total measurements from 5 cells were best fit using a two-component model with brightness values constrained to 9500 and 17000 representing monomer and dimer, respectively (*χ* ^2^ = 1.04). Of 75 total measurements included in the analysis, dimer was detected in 52 measurements and in 42 measurements >10% of the total particles detected represent dimer (Figure 5(b)). Among the 52 measurements that detect dimer, the average concentration of monomer is 19.81 ± 2.40 nM and the average concentration of dimer is 3.99 ± 0.06 nM. On average, 20 ± 0.3% of the particles detected are dimer, indicating that 28.7% of AKAP7*γ*-EGFP is in the dimeric form. Based upon the average concentrations of monomer and dimer, the apparent Kd of dimerization falls in the range of 78.8–126 nM with an average of 98.4 nM, consistent with the kinetic data from SPR. Using quantitative western blot for AKAP7*γ* in human aortic smooth muscle cells, we were able to estimate that the average concentration per cell is approximately 180 nM (Figure 5(c)). Since the cellular concentration is roughly twice the Kd, we expect a significant amount of dimerization* in vivo*. Attempts to detect higher order oligomeric structures by fitting AKAP7*γ*-EGFP data with larger brightness values were unsuccessful. However, this does not exclude the possibility that higher order AKAP7*γ* oligomers exist. The concentrations of higher order oligomers were likely insufficient either for detection or for discrimination from the population of monomers and dimers. This evidence offers further confirmation that self-association of AKAP7*γ* occurs in live cells.

### 3.6. Computational Modeling Predicts That Oligomerization of AKAP7*γ* May Function to Increase Target Phosphorylation

**Figure 6:**
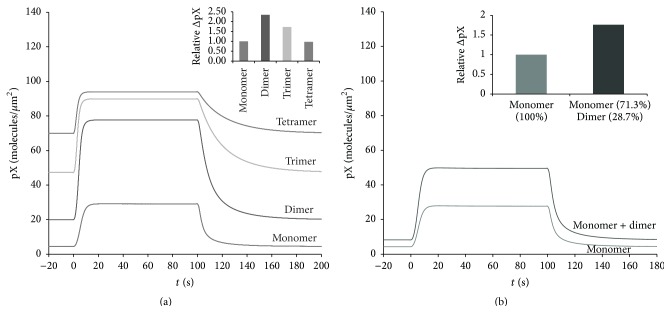
Computational modeling predicts that oligomerization of AKAP7*γ* may function to increase target phosphorylation. (a) Time course of target protein X phosphorylation for each oligomeric state. An increase in cAMP production is simulated from 0 to 100 s resulting in activation of AKAP bound PKA and phosphorylation of target protein X. The inset shows the change in [pX] from baseline to maximal phosphorylation relative to the change in phosphorylation produced by the monomeric state. (b) Time course of target X phosphorylation for a mixture of monomer and dimer as measured via PCH. The concentration of monomeric and dimeric AKAP complexes was adjusted so that 71.3% of the total AKAP exists as monomer and 28.7% exists as dimer. An increase in cAMP production was simulated as above. The inset shows the change in [pX] from baseline to maximal phosphorylation relative to 100% monomer.

Our finding that AKAP7*γ* is able to form homodimers is interesting and leads us to question the functional role of this oligomerization. The two large regions of interaction make it exceedingly difficult to disrupt AKAP7*γ* oligomerization. Thus, we decided to test the possible functional significance of AKAP7*γ* oligomerization* in silico*. We created a compartmental deterministic model that simulates the dynamics of the PKA regulatory (RII*α*) and catalytic subunits (C*α*) in response to cAMP, both in the cytosol and on the endoplasmic reticulum (ER) membrane. We chose to model the dynamics of AKAP7*γ* at the ER due to recent work demonstrating localization of the scaffold at the ER in cardiac myocytes [16]. Included in the model is autophosphorylation of RII*α*, as this is documented as a key component for activation of C*α* when complexed with RII*α* [28, 32, 33]. In our model, it is assumed that the probability of activation of all C*α* subunits within an oligomer increases as the total number of activated C*α* subunits increases ((1), Section 2). Active C*α* subunits were modeled to participate in the phosphorylation of a generic target protein X (X → pX) existing on the ER membrane ((2), Section 2). The time course of formation of pX is shown for separate simulations that compare oligomeric states from monomer to tetramer (Figure 5(a)). Upon stimulation of PKA activity, the amount of pX increases in all cases. When the monomeric state (*n* = 1) is simulated, phosphorylation of our target protein occurs as it would if the feed-forward assumption is not made. Removal of the stimulus leads to dephosphorylation of pX and return to baseline. Oligomerization of AKAP7*γ* affects the baseline, magnitude, and speed of phosphorylation of protein X (Figures 6(a) and 6(b)). The largest magnitude change of pX occurred in the dimeric state, with larger order AKAP7*γ* oligomers exhibiting decreased magnitude change in pX (Figure 6(b)). When the active scaffolded PKA population was adjusted to reflect a mixture of monomeric and dimeric states according to our PCH data, the baseline phosphorylation level remains low (8.26 molecules/*μ*m^2^) while the magnitude change in pX increases by 76% in comparison to monomer only (Figures 6(a) and 6(b)). This analysis demonstrates the potential impact of AKAP7*γ* oligomerization on the rate of PKA substrate phosphorylation.

## 4. Discussion

Significant work has demonstrated the impact of scaffolding on the organization of signaling proteins into discrete focal points of subcellular enzyme activity [34]. However, information regarding the molecular architecture of scaffolds and its influence on phosphorylation events is currently lacking. Here, through the use of a combination of protein biochemistry, fluorescence correlation spectroscopy, and computational modeling, we deciphered that the scaffolding protein AKAP7*γ* dimerizes* in vitro* and in cells. Our ability to detect dimerization via PCH indicates that dimerization is occurring in live cells. Importantly, computational modeling suggests that dimerization may act to increase target phosphorylation. These findings represent a previously unappreciated mechanism involved in the configuration of AKAP complexes and the regulation of protein phosphorylation.

Several recent reports have demonstrated homooligomerization of AKAP5, AKAP12, and AKAP-Lbc [11, 12, 14]. Dimer formation of these AKAPs is mediated by direct protein-protein interactions. A leucine zipper motif in the N-terminus of AKAP-Lbc dictates the interaction while multiple domains of AKAP5 are required for dimerization [11, 12]. As previously shown, AKAP7*γ* dimerization also occurs via direct interaction. Our efforts to map the sites of binding indicate that two regions of interaction exist. Interestingly, the crystal structure of the common central domain contained in the long AKAP7 isoforms (amino acids 76–292 of AKAP7*δ*) does not indicate oligomerization of the AKAP [35]. This structure lacks the N-terminus of AKAP7*γ*, which is predicted to exist in an unordered arrangement, according to FOLDindex^©^. Several AKAPs have a natively disorganized, unfolded configuration that is hypothesized to aid in the docking of a multitude of binding partners [12, 14, 35]. However, this unstructured flexibility also significantly hinders conventional structural analysis. Hence, this N-terminal domain of AKAP7*γ* may contribute significantly to its oligomerization. In fact, our peptide array analysis demonstrates that the majority of the interacting sequence from 43 to 98 is not contained in the original crystal structure. Other possible mechanisms include posttranslational modification driven interactions and coiled-coil interactions [36–38]. Analysis of the AKAP7*γ* sequence using the PredictProtein website as well as both MARCOIL and LOGICOIL web resources supports these possibilities for AKAP7*γ*. Testing these potential mechanisms for oligomerization will help in delineating the means of AKAP7*γ* complex formation.

Signaling proteins utilize oligomerization to enhance their known cellular functions. However, the consequences of AKAP oligomerization are still unclear but may impact multiple processes. Mass spectrometry analysis of the AKAP5 complex suggests that, upon dimerization, AKAP5 associates with four calcineurin phosphatase heterodimers and two calmodulin molecules [12]. By increasing the concentration of the phosphatase in a localized area, it is suggested that AKAP5 can generate specific pools of second messenger-mediated events at the plasma membrane. How this change in stoichiometry affects substrate dephosphorylation or kinetics of enzyme activation has not been investigated. However, other studies of the native AKAP5 complex in hippocampal neurons suggest that this increase in stoichiometry is an* in vitro* artifact, as no increase in stoichiometry was detected* in vivo* [39]. Oligomerization may also affect regulation of intrinsic enzymatic activity of an AKAP. AKAP-Lbc is a guanine nucleotide exchange factor (GEF) for RhoA [40]. Disruption of AKAP-Lbc dimerization dramatically increased its GEF activity, suggesting that oligomer formation acts to limit the basal activity of the AKAP [11]. However, AKAP7*γ* does not display any known intrinsic enzymatic activity, suggesting that this mechanism is not applicable.

**Figure 7:**
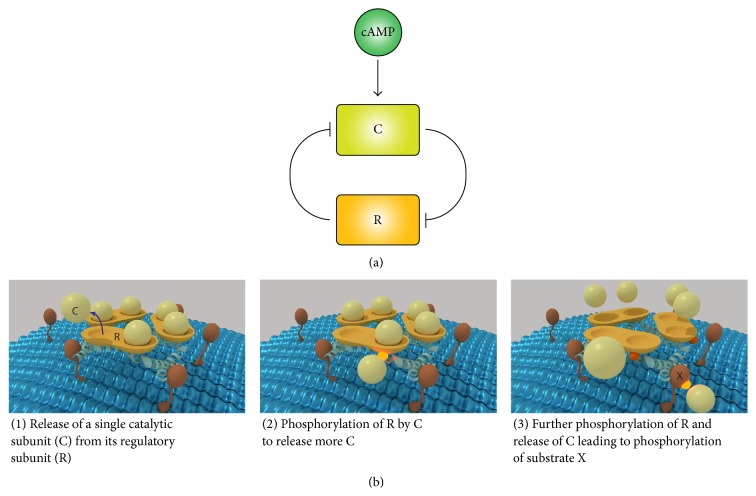
Feed-forward model of PKA activation. (a) Double-negative feedback loop. In the resting state, C*α* is inhibited by RII*α*. However, once C*α* is activated by cAMP it is able to phosphorylate RII*α* at Ser-99, which prevents RII*α* from inhibiting C*α*. (b) Illustration of the feedback mechanism occurring within a hypothetical trimeric AKAP complex. AKAP (light blue helix) recruits PKA (R homodimer, orange; C, light yellow) to a membrane associated target X (red). In the context of this AKAP oligomer, activation of a single C*α* is likely to phosphorylate RII*α* within the complex leading the release of further C*α*. This process increases the probability that all C*α* within the complex will become active as each additional C*α* is activated. The increased amount of active C*α* also increases the probability of target phosphorylation.

To explore the possible functional significance of AKAP7*γ* oligomerization, we created a computational model of PKA scaffolding. Significant work has demonstrated that release of active C*α* subunit from the PKA holoenzyme requires not only cAMP binding to the RII*α* subunit but also autophosphorylation of RII*α* [28, 32, 33]. This leads us to hypothesize that, in the context of an AKAP7*γ* oligomer, release of a single C*α* subunit can potentiate the release of additional C*α* via phosphorylation of RII*α* present in the oligomer. In the monomeric state, a single C*α* can stimulate release of only one extra C*α* subunit, but, in a dimeric or oligomeric state, release of additional C*α* subunits is possible. This feedback loop ultimately increases substrate phosphorylation due to the increased probability of phosphorylation resulting from increased C*α* release (Figure 7). This is simulated by defining activation of PKA in the AKAP7*γ* complex as a probability function in which the chance of activating all C*α* subunits increases as the number of active C*α* subunits within the complex increases (1). The model suggests that the physiologic consequence of AKAP7*γ* oligomerization could be increased baseline phosphorylation, increased magnitude of phosphorylation in comparison to the monomeric state, and increased speed of phosphorylation. For the distribution of monomeric and dimeric AKAP7*γ* derived from our PCH experiment, the model predicts a 1.91-fold increase in baseline phosphorylation and a 1.77-fold increase in magnitude of phosphorylation in comparison to monomer only. It is important to note that our finding does not result from an increase in AKAP7*γ* or PKA concentrations at the SR membrane; it reflects the consequence of oligomerization on PKA activity. In all simulations the total amount of PKA scaffolded to the membrane by AKAP7*γ* remains the same. The feed-forward behavior of C*α* activation has the potential for interesting consequence when considered in a spatial context. The free diffusion of active C*α* may permit its interaction with AKAP7*γ* complexes that exist beyond the location of its complex of origin. This could enable the spatial spread of PKA activation and target phosphorylation similar to that of a calcium spark. Spatial spread of the PKA signal in this manner would depend upon many factors including the diffusion coefficient of C*α*, the density of AKAP complexes, the ability of C*α* to reassociate with RII*α*, and the spatiotemporal fluctuation in cAMP concentration.

The importance of AKAP7 to PLB phosphorylation and cardiac calcium dynamics has recently been called into question. Jones and colleagues created a mouse expressing a truncated AKAP7, consisting of deletion of exon 7 of the* AKAP7* gene [41]. This exon transcribes the PKA binding domain common to all AKAP isoforms; thus these mice are expected not to have PKA localized to the complex. However, they had no defects in *β*-AR-stimulated phosphorylation of phospholamban. Hence, the authors suggest that AKAP7 is not responsible for anchoring PKA to phospholamban and suggest that another AKAP likely performs this function. Their deletion construct is expected to ablate only the PKA and Ca_v_1.2 binding domains of all AKAP7 isoforms, although no effect on L-type calcium current was seen. Additionally, as exons 1–6 of AKAP7*γ* are still expressed in the mouse, leaving a large, truncated protein that would bind all the other components (phospholamban, PP1, I-1, PDE4D3, and PDE4D3), it is noteworthy that the authors did not demonstrate that the truncated protein products do not associate with PKA. If a truncated version of AKAP7*δ*/*γ* is still expressed in the mouse, it would still bind all the other components of the complex and perhaps the balance of phosphorylation to dephosphorylation is changed. In an investigation that compared the effects of a complete knockout of AKAP5 versus the one that just deleted the PKA binding domain, significant decreases in substrate phosphorylation were only seen in the complete knockout [42]. This was due to the impact of the other scaffold proteins that still associated with the truncated AKAP5 protein. Hence, the other binding partners of AKAP7*δ*/*γ* that should still associate with the truncated protein may have a dramatic effect on phospholamban phosphorylation. Further investigation of this mouse model is warranted before concluding that AKAP7*δ*/*γ* anchoring to PKA is not important.

## 5. Conclusion

We have discovered an additional component of the AKAP7*γ* signaling complex and predicted via computational modeling that dimerization of AKAP may impact phosphorylation of PKA substrates. As several PKA targets of AKAP7*γ* have been identified, including PDE4D, PDE4D3, the PP1 inhibitor I-1, and the cardiac calcium regulatory protein phospholamban, AKAP7*γ* oligomerization has the possibility of affecting phosphorylation of these endogenous substrates.

## Supplementary Material

Reaction Kinetic parameters for PKA dynamics used in the model are shown in the Supplemental Table 1.
The network diagram designed to test our model is shown in Supplemental Figure 1.
Supplemental Figure 2: AKAP7*γ* does not interact with any other AKAPs expressed in the heart. HEK293 cells were transfected with either mAKAP-EGFP or AKAP79-EGFP. Isolated cell lysates were subjected to pulldown assays with either AKAP7*α*-S-Tag or AKAP7*γ*-S-Tag before subjected to Western blot analysis to determine association.

## Abbreviations

AKAP: A-Kinase anchoring protein
SPR: Surface plasmon resonance
PCH: Photon counting histogram
MBP: Maltose binding protein
PKA: cAMP-dependent protein kinase
MAP: Mitogen-activated protein.
