# Supplementary material for: Analysis of AKAP7γ Dimerization
Source: J Signal Transduct. 2015 Aug 31;2015:371626. doi: 10.1155/2015/371626 (PMC4568377; doi:10.1155/2015/371626)

Reaction	Kinetics	kcat	Km	Kf	Kr	Reference
ATP --> cAMP	Henri-Michaelis-Menten	12 s^{-1}	11 μm			BRENDA Database
X --> pX	General	22.6 s^{-1}	65.2 molecules/μm^2			Masterson et. Al 2011
pX --> X	Henri-Michaelis-Menten	2.4 s^{-1}	65.0 molecules/μm^2			BRENDA Database
cAMP --> AMP (cyt)	Henri-Michaelis-Menten	2.0 s^{-1}	5.5 μm			BRENDA Database
cAMP --> AMP (mem)	Henri-Michaelis-Menten	0.098 s^{-1}	5.5 μm			
R + cAMP --> RA	Mass Action			100 $\mu m^{-1} s^{-1}$	2 s^{-1}	
C + R --> RC	Mass Action			0.31 $\mu m^{-1} s^{-1}$	0.00023 s^{-1}	Diskar 2010
RC + cAMP --> C + RA	Mass Action			1 $\mu m^{-1} s^{-1}$	0.177 $\mu m^{-1} s^{-1}$	Diskar 2010
AKAP_RC + cAMP --> AKAP_R + C_mem	Mass Action			1 $\mu m^{-1} s^{-1}$	0.177 $\mu m^{-1} s^{-1}$	Diskar 2010
AKAP + RC --> AKAP_RC	Mass Action			1 $\mu m^{-1} s^{-1}$	0.005 s^{-1}	Herberg et. al. 2000
PKI + C_mem --> PKI_C	Mass Action			1 $\mu m^{-1} s^{-1}$	0.01 s^{-1}	

Supplemental Figure 1

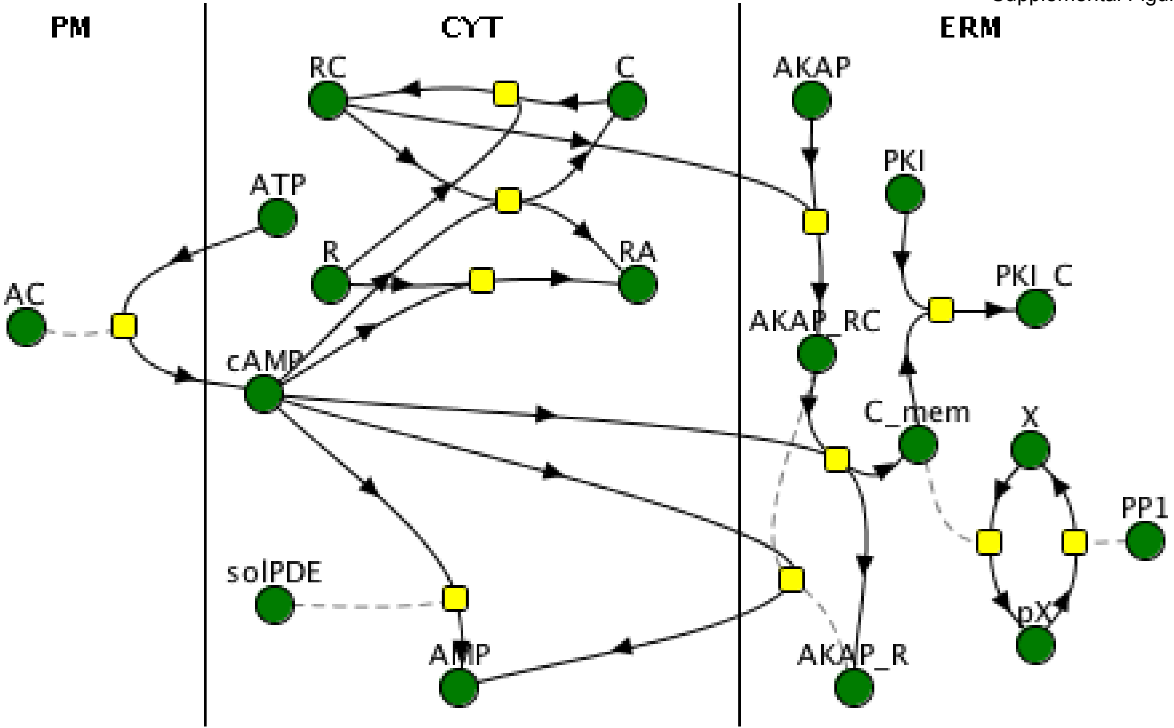

Supplemental Figure 2

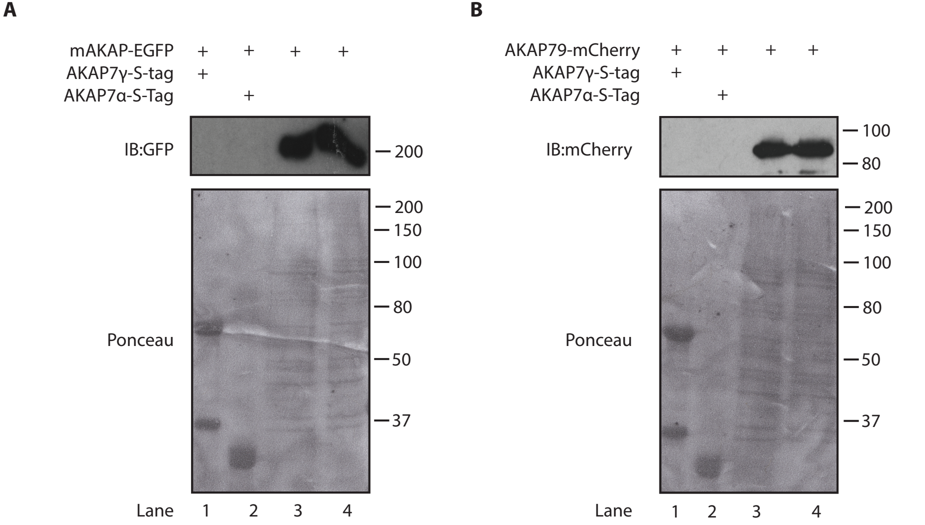

Supplement: Supplementary file 1 — Reaction Kinetic parameters for PKA dynamics used in the model are shown in the Supplemental Table 1. The network diagram designed to test our model is shown in Supplemental Figure 1. Supplemental Figure 2: AKAP7γ does not interact with any other AKAPs expressed in the heart. HEK293 cells were transfected with either mAKAP-EGFP or AKAP79-EGFP. Isolated cell lysates were subjected to pulldown assays with either AKAP7α-S-Tag or AKAP7γ-S-Tag before subjected to Western blot analysis to determine association. [file 371626.f1.pdf]
